# Predictors of short- and long-term mortality in critically ill, older adults admitted to the emergency department: an observational study

**DOI:** 10.1186/s12873-022-00571-2

**Published:** 2022-01-27

**Authors:** Henrik Olsson, Björn W. Karlson, Johan Herlitz, Thomas Karlsson, Jenny Hellberg, Mattias Prytz, Ninni Sernert, Niklas Ekerstad

**Affiliations:** 1 Department of Cardiology, NU Hospital Group, Trollhättan, Sweden; 2 Department of Molecular and Clinical Medicine, Institute of Medicine, Sahlgrenska Academy, University of Gothenburg, Gothenburg, Sweden; 3 AstraZeneca Gothenburg, Mölndal, Sweden; 4 Center for Prehospital Research, Faculty of Caring Science, Work Life and Social Welfare, University of Borås, Borås, Sweden; 5 Biostatistics, School of Public Health and Community Medicine, Institute of Medicine, Sahlgrenska Academy, University of Gothenburg, Gothenburg, Sweden; 6 Department of Surgery, NU-Hospital Group, Region Västra Götaland, Trollhättan, Sweden; 7 Department of Orthopaedics Sahlgrenska Academy, Institute of Clinical Sciences, University of Gothenburg, Gothenburg, Sweden; 8 Department of Gothenburg Health, Medicine and Caring Sciences, Unit of Health Care Analysis, Linköping University, Linköping, Sweden

**Keywords:** Older adults, Emergency department, Predictors, Mortality

## Abstract

**Background:**

In the future, we can expect an increase in older patients in emergency departments (ED) and acute wards. The main purpose of this study was to identify predictors of short- and long-term mortality in the ED and at hospital discharge.

**Methods:**

This is a retrospective, observational, single-center, cohort study, involving critically ill older adults, recruited consecutively in an ED. The primary outcome was mortality. All patients were followed for 6.5–7.5 years. The Cox proportional hazards model was used.

**Results:**

Regarding all critically ill patients aged ≥ 70 years and identified in the ED (*n* = 402), there was a significant association between mortality at 30 days after ED admission and unconsciousness on admission (HR 3.14, 95% CI 2.09–4.74), hypoxia on admission (HR 2.51, 95% CI 1.69–3.74) and age (HR 1.06 per year, 95% CI 1.03–1.09), (all *p* < 0.001).

Of 402 critically ill patients aged ≥ 70 years and identified in the ED, 303 were discharged alive from hospital. There was a significant association between long-term mortality and the Charlson Comorbidity Index (CCI) > 2 (HR 1.90, 95% CI 1.46–2.48), length of stay (LOS) > 7 days (HR 1.72, 95% CI 1.32–2.23), discharge diagnosis of pneumonia (HR 1.65, 95% CI 1.24–2.21) and age (HR 1.08 per year, 95% CI 1.05–1.10), (all *p* < 0.001). The only symptom or vital sign associated with long-term mortality was hypoxia on admission (HR 1.70, 05% CI 1.30–2.22).

**Conclusions:**

Among critically ill older adults admitted to an ED and discharged alive the following factors were predictive of long-term mortality: CCI > 2, LOS > 7 days, hypoxia on admission, discharge diagnosis of pneumonia and age. The following factors were predictive of mortality at 30 days after ED admission: unconsciousness on admission, hypoxia and age. These data might be clinically relevant when it comes to individualized care planning, which should take account of risk prediction and estimated prognosis.

## Background

Worldwide, there is a large and growing group of older adults [1], many with co-morbidities. This trend implies increasing healthcare needs, which will have an impact on the healthcare, social and financial systems in all countries in the future [2]. The absolute numbers of visits and rate of visits per population unit have increased in emergency departments (EDs) [3, 4]. The healthcare needs of older adults are largely responsible for this trend [4, 5]. In the future, we can expect an even more substantial increase in older patients in the ED and acute wards [6]. Older patients with multiple chronic diseases represent a large proportion of frequent ED users [7].

Older adults consume more ED resources, are more frequently admitted to a hospital ward and stay longer compared with younger patients [4, 8]. They also seek care in the ED for several different reasons at the same time and present heterogeneous patterns of morbidity. These patients might be more vulnerable [2, 9], and they run a higher risk of adverse health outcomes [10]. Moreover, they run a higher risk of death, ED revisits, hospitalizations, functional decline and loss of independence within a short period of time compared with younger patients [4, 6, 11, 12]. Geriatric patients are not solely defined by age, but are instead characterized by the presence of acute and chronic diseases combined with age-related changes [6]. Several studies have demonstrated that the influence of co-morbidities on the prognosis is important [11, 13].

### Importance

The benefit/risk ratio of different interventions for a patient might be influenced by the estimated prognosis. The early identification of risk factors for mortality and other adverse outcomes in older adults on admission to the ED and at subsequent discharge from hospital could provide valuable information on ways of preventing future events, individualizing interventions and making informed treatment decisions [2, 6, 14], e.g. the need for a comprehensive geriatric assessment (CGA) [6, 15, 16]. Predictors of poor in-hospital and short-term prognosis in older patients have been described in previous studies [1, 2, 17, 18]. However, markers for making a prognosis of long-term outcomes in older adults in EDs and acute wards are scarce [17, 19]. The co-morbidity burden may have a substantial impact on long-term mortality, particularly in older adults.

### Aims

1. To describe a cohort of consecutive critically ill older ED patients (aged ≥ 70) regarding characteristics and outcomes in terms of short- and long-term mortality, and mid-term re-hospitalizations and number of hospitalization days
2. To identify prognostic markers available in the ED and at discharge regarding short- and long-term mortality

## Methods

### Study design and setting

This is a retrospective, observational, single-center cohort study. It includes critically ill older adults, recruited at the ED at the Northern Älvsborg-Uddevalla (NU) Hospital Group, Region Västra Götaland, Sweden, between February 2013 and February 2014. This county hospital has an uptake population of approximately 280 000 inhabitants.

The study was conducted in accordance with the Declaration of Helsinki and Good Clinical Practice Guidelines, and was approved by the regional ethical review board at Sahlgrenska University Hospital in Gothenburg, Sweden (D.no. 962–13). The study was registered at the Swedish National Database of Research and Development; identifier 142,071 (http://www.researchweb.org/is/vgr/project/142071; February 5, 2014) as Medical Emergency Care (MEC)—an observational study of the emergency care of the critically ill medical patient. Before a secondary data collection regarding long-term mortality was performed, complementary ethical approval was given by the Swedish ethical review authority (D.no. 2020–04,407), waiving the need for a renewal of the informed consent. Due to the expected high mortality rate it would not have been possible to collect a second informed consent.

### Data collection and participants

The primary data collection has been described previously in Bergh et al. [20] All adult internal medicine patients treated in the ED and classified as critically ill in accordance with the Rapid Emergency Treatment Triage System (RETTS) [21] were included consecutively. RETTS, developed for risk assessment in EDs, has been used in order to perform a sensitive identification of critically ill patients [22, 23]. It relies on the following vital signs (VS): airway obstruction/stridor; oxygen saturation < 90%; respiratory rate < 8 or > 30 per minute; regular heart rhythm > 130 or irregular heart rhythm > 150 beats per minute; systolic blood pressure < 90 mmHg; unconsciousness, defined as Reaction Level Scale (RLS) > 3 or Glasgow Coma Scale (GCS) < 8; ongoing seizure [20, 22, 23]. Simultaneously the symptoms that caused the contact with health care is to be considered (the Emergency Signs and Symptoms code [ESS code]). The combination of VS and ESS gives the patient a colour of either red, orange, yellow, green or blue in order of severity of the condition and reflecting the time required to assessment by a physician. In this study we included patients given the colour red, reflecting urgent requirement of a physician assessment, i.e. critically ill patients.

The exclusion criteria were lack of written informed consent, if a patient was wrongly registered, and if the patient was treated for cardiac arrest, need for acute percutaneous coronary intervention (PCI) or included in the acute stroke fast track [20]. Patients with trauma or other surgical conditions were excluded. Information was collected retrospectively from the ambulance records and the hospital medical records.

A secondary data collection was performed regarding mortality until December 31, 2020. This information was extracted from the State’s Personal Address Register (SPAR) at the Swedish Tax Agency. This is a comprehensive state agency register, which includes all persons who are registered as residents in Sweden. The data in SPAR are updated every day with data from the Swedish Population Register [24], and are a reliable source of information regarding death and survival confirmation.

Approximately 7% of all internal medicine patients ≥ 70 years of age admitted to the ED were critically ill. Of 832 patients correctly classified as critically ill, written informed consent was given by 610 patients [20]. For the analyses described here, patients aged ≥ 70 years were selected.

### Methods and measurements

Clinical and demographic characteristics were primarily collected at index admission to the ED from the patient ambulance records and subsequent medical records from the ED and the hospital medical wards. The following variables were recorded: age, sex, date and time of arrival at the ED, main symptoms and VS in the ambulance, working diagnosis in the ED and medical history including the Charlson Comorbidity Index (CCI) components. At discharge from hospital, the type of department to which the patient was admitted, care in the intensive care unit (ICU) or cardiac intensive care unit (cICU), length of stay (LOS) in hospital, discharge diagnosis and in-hospital mortality were recorded.

Data regarding post-discharge outcomes up to 12 months were collected from medical records. These included information on mortality, re-hospitalizations and total LOS. A secondary data collection was performed regarding mortality, in which all patients were followed-up for 6.5–7.5 years post-discharge, as described in the data collection section. The cases refer to unique individual patients, and re-hospitalizations were registered as an outcome.

#### The Charlson Comorbidity Index

The patient’s total burden of morbidity was measured by the CCI [25, 26]. It contains 19 categories of comorbidity and predicts mortality for a patient in a general medical context. Each comorbidity is assigned a score of 1, 2, 3, or 6, depending on the risk of death associated with this condition.

The CCI score was dichotomized as 0–2 (mild grade) versus > 2 (moderate or severe grade), a commonly applied stratification [27–29].

### Outcomes

The primary outcome was all-cause post-discharge death until December 31, 2020.

Secondary outcomes were death within one month after admission to the ED; and total time spent in hospital and numbers of re-hospitalizations up to 12 months post-discharge.

### Statistical analysis

Descriptive statistics are presented as number and percentage, mean ± standard deviation or median with 25th, 75th percentiles. A Cox proportional hazards model was used to calculate hazard ratios (HR) and corresponding 95% confidence intervals regarding 30-day and long-term mortality, in both univariable and multivariable analyses.

To identify independent predictors of mortality, we first used stepwise backward selection, starting with a model including age and all the other candidate variables with an un adjusted p-value below 0.30 and using *p* < 0.05 as the limit for staying in the model. After this selection procedure was finished, we included all the remaining variables with an age adjusted *p* < 0.30 separately, one at a time, to see whether they contributed significantly to the model (using differences in -2 log likelihood). The above was performed separately for 30-day and long-term mortality respectively.

The Kaplan–Meier (KM) method was used to calculate cumulative mortality curves, using 100—KM survival estimate as an assessment of cumulative incidence. This method was also used for calculation of rehospitalization rate during the first 12 months for patients discharged alive after index, where those non-rehospitalized who died were censored at time of death and comparisons between CCI groups were performed using the log rank test.

Numbers of re-hospitalizations and total days rehospitalized during 12 months after index discharge among those alive at 12 months were compared between CCI groups using the Mann–Whitney U test.

All tests were two-sided and p-values below 0.05 were considered statistically significant. All analyses were performed using SAS for Windows version 9.4.

## Results

**Fig. 1:**
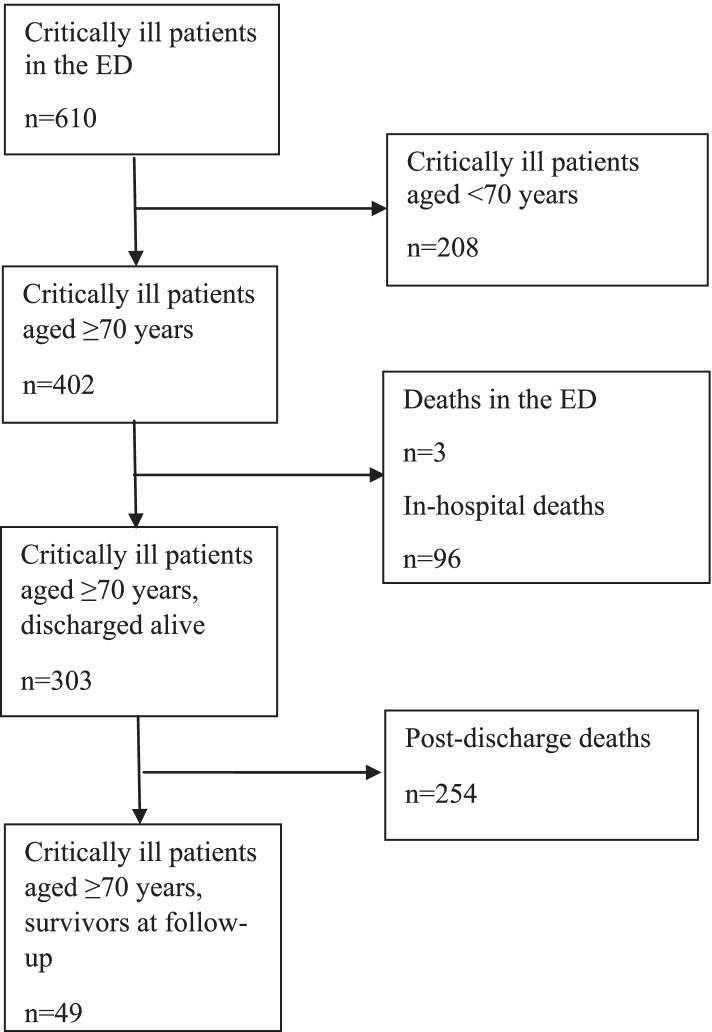
Flow chart

Of 610 critically ill patients identified in the ED, 402 were aged ≥ 70 years. Of these, three patients (0.7%) died in the ED and six (1.5%) were able to return home directly from the ED. There were 96 (23.9%) in-hospital deaths. Of the 303 patients discharged alive, directly from the ED or from a hospital ward, 254 (83.8%) died before the end of follow-up (on December 31, 2020), see flow chart, Fig. 1.

### Short-term mortality

The baseline characteristics of the patients aged ≥ 70 years (*n* = 402) are shown in Table 1. Their mean age was 82.1 years (SD 6.4) and 221 (55.0%) were male. They had a large comorbidity burden, the most common conditions being cardiovascular disease, diabetes, chronic obstructive pulmonary disease (COPD) and dementia. Regarding the CCI, 208 patients (51.7%) scored 1–2, 85 (21.1%) scored 3–4 and 41 (10.2%) scored > 4. On admission, the most commonly reported main symptoms were dyspnea, an episode of unconsciousness, chest pain, seizure and vomiting. Regarding vital signs on admission, 224 (56.4%) had hypoxia, 215 (59.9%) had a respiratory rate of < 8 or > 30, 142 (35.3%) showed signs of infection, 82 (20.4%) had tachycardia, 65 (16.2%) were unconscious, 52 (13.0) were hypotensive, 26 (6.5%) suffered from obstructive airways and 15 (3.7%) presented with seizure. Approximately half of the patients were admitted during office hours. The five most common main discharge diagnoses were pneumonia, heart failure, urosepsis, COPD and atrial fibrillation. Regarding hospital care level, 187 patients (46.5%) were admitted to a conventional medical ward, 130 (32.3%) to a medical emergency ward and 76 (18.9%) to the ICU/cICU. The mean LOS was 10.3 days (SD 8.9).

**Table 1:** Baseline characteristics of critically ill patients aged ≥ 70 years admitted to the ED (*n* = 402)

Variable, n (%)	
Demographics	
Age, years, mean (SD)	82.1 (6.4)
Male sex, n (%)	221 (55.0)
Medical history n (%)	
CCI score, n	
0	68 (16.9)
1–2	208 (51.7)
3–4	85 (21.1)
> 4	41 (10.2)
IHD	80 (19.9)
CHF	92 (22.9)
PAD	27 (6.7)
CVD	92 (22.9)
Dementia	70 (17.4)
COPD	88 (21.9)
Diabetes	91 (22.6)
without chronic complications	73 (18.2)
with chronic complications	18 (4.5)
Chronic kidney diseasea	36 (9.0)
Malignant disease	46 (11.4)
without metastases	26 (6.5)
metastatic solid tumor	12 (3.0)
lymphoma	7 (1.7)
leukemia	1 (0.2)
Main reason for admissionb n (%)	
Dyspnea	200 (49.8)
Unconsciousness	59 (14.7)
Chest pain	46 (11.4)
Seizure attack	16 (4.0)
Vomiting	22 (5.5)
Vital signs on admission	
Obstructive airway, n (%)	26 (6.5)
Hypoxiac, n (%) (5)d	224 (56.4)
Hypotensione, n (%) (3)	52 (13.0)
Respiratory rate (br/min), ≤ 8/ ≥ 30, n (%) (43)	215 (59.9)
Heart rate (bpm) ≥ 130/ ≥ 150f(1)	82 (20.4)
RLS > 3, n (%)	65 (16.2)
Ongoing seizures, n (%)	15 (3.7)
Signs of infection, n (%)	142 (35.3)
Admission time point	
Workday 8 am – 8 pm	195 (48.5)
Main index discharge diagnosisg n (%)	
Pneumonia	85 (21.1)
Heart failure	33 (8.2)
Atrial fibrillation	21 (5.2)
COPD	27 (6.7)
Urosepsis	29 (7.2)
Hospital care level n (%)	
Intensive care unit or cardiac intensive care unit	76 (18.9)
Medical emergency ward	130 (32.3)
Other wards	187 (46.5)
Not hospitalized	6 (1.5)
Deceased at emergency department	3 (0.7)
LOS, index, mean (SD)(n)	10.3 (8.9)

*CCI* Charlson Comorbidity Index, *IHD* ischemic heart disease, *CHF* congestive heart failure, *PAD* peripheral arterial disease, *CVD* cerebrovascular disease, *COPD* chronic obstructive pulmonary disease, *br/min* breaths per minute, *bpm* beats per minute, *LOS* length of stay

^a^Moderate or severe renal disease. Severe = on dialysis, status post kidney transplant, uremia, moderate = creatinine > 3 mg/dL (0.27 mmol/L)

^b^Five most commonly reported main symptoms in the ambulance

^c^Oxygen saturation < 90%

^d^Number missing

^e^Systolic blood pressure < 90 mmHg

^f^Regular/irregular

^g^Five most common diagnoses

The association between baseline characteristics possible to obtain on admission to the ED (thus excluding hospital care level, LOS and discharge diagnosis) (*n* = 402) and all-cause mortality until 30 days after admission to the ED is presented as unadjusted HRs in Table 2. There were 125 deaths. The following variables were significantly associated with 30-day mortality: unconsciousness, hypoxia, RLS > 3 and age (all *p* < 0.05).

**Table 2:** Unadjusted analysis regarding death within 30 days after ED admission for critically ill patients aged ≥ 70 (*n* = 402)

Variable	Prevalence n (%)	Unadjusted HR (95% CI)	*p*-value
CCI > 2	126 (31.3)	1.06 (0.73–1.54)	0.76
Age, mean (SD)	82.1 (6.4)	1.06 (1.03–1.09)	< 0.0001
Female sex	181 (45.0)	0.94 (0.66–1.34)	0.72
ICU/cICU	76 (18.9)	0.83 (0.51–1.34)	0.44
Symptoms on admissiona			
Dyspnea	200 (49.8)	1.14 (0.80–1.62)	0.47
Unconsciousness	59 (14.7)	2.61 (1.74–3.90)	< 0.0001
Chest pain	46 (11.4)	0.57 (0.29–1.12)	0.11
Seizure attack	16 (4.0)	0.17 (0.02–1.19)	0.07
Vomiting	22 (5.5)	0.50 (0.18–1.35)	0.17
Vital signs on admission			
Obstructive airway	26 (6.5)	1.71 (0.92–3.18)	0.09
Hypoxiab (5)c	224 (56.4)	2.23 (1.51–3.30)	< 0.0001
Hypotensiond (3)	52 (13.0)	0.84 (0.48–1.46)	0.54
Respiratory rate (br/min), ≤ 8/ ≥ 30 (43)	215 (59.9)	1.45 (0.98–2.14)	0.06
Heart rate (bpm) ≥ 130/ ≥ 150e (1)	82 (20.4)	0.56 (0.34–0.93)	0.03
RLS > 3	65 (16.2)	2.43 (1.64–3.61)	< 0.0001
Ongoing seizure attack	15 (3.7)	0.38 (0.09–1.52)	0.17
Signs of infection	142 (35.3)	0.75 (0.51–1.09)	0.13
Admission time point			
Workday 8 am—8 pm	195 (48.5)	1.12 (0.78–1.58)	0.54

Four hundred two patients were included in the analysis. There were one hundred twenty-five deaths

*CCI* Charlson Comorbidity Index, *ICU* intensive care unit, *cICU* coronary intensive care unit, *br/min* breaths per minute, *bpm* beats per minute, *RLS* Reaction Level Scale

^a^Five most commonly reported main symptoms in the ambulance

^b^Oxygen saturation < 90%

^c^Number missing

^d^Systolic blood pressure < 90 mmHg

^e^Regular/irregular

The following variables were identified as independent predictors of 30-day mortality, presented in order in terms of magnitudes of the HRs: unconsciousness on admission (HR 3.14, 95% CI 2.09–4.74), hypoxia on admission (HR 2.51, 95% CI 1.69–3.74) and age (HR 1.06 per increasing year, 95% CI 1.03–1.09), (all *p* < 0.001), see Table 3**.**

**Table 3:** Multivariable analysis of predictors of death within 30 days after ED admission for critically ill patients aged ≥ 70 (*n* = 402)

**Variable**	**Multivariable** **HR (95% CI)**	***p*-value**
Age; per year	1.06 (1.03–1.09)	0.0002
Symptoms on admission Unconsciousness	3.14 (2.09–4.74)	< 0.0001
Vital signs on admission Hypoxia	2.51 (1.69–3.74)	< 0.0001

Four hundred two patients were included in the analysis. There were one hundred twenty-fivedeaths

### Long-term mortality

**Fig. 2:**
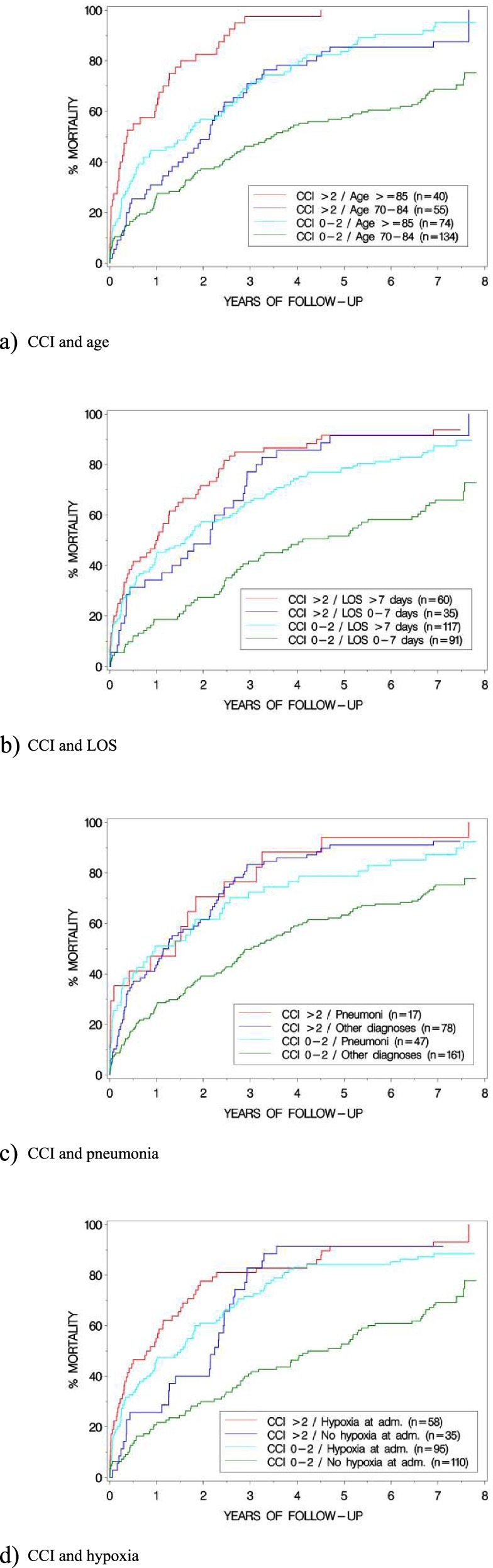
Kaplan Meier (KM) estimated cumulative mortality

The characteristics of critically ill patients aged ≥ 70 years who were discharged alive directly from the ED [*n* = 6] or from index hospitalization [*n* = 297] (*n* = 303) are shown in Table 4. The association between these characteristics and all-cause mortality until December 31, 2020 is presented as unadjusted HRs. There were 254 deaths. The following variables were significantly associated with long-term mortality: hypoxia on admission, CCI score > 2, LOS > 7 days, respiratory rate < 8 or > 30 on admission, diagnosis of pneumonia at discharge, dyspnea on admission, age and admission during workday time (8 am until 8 pm) (all *p* < 0.05). KM estimated cumulative mortality is reported in Fig. 2, in which a CCI of > 2 was combined with each of the other independent predictive factors from the multivariable analysis (see below), respectively**.** There was no significant interaction between the CCI and any of these other variables.

**Table 4:** Unadjusted analysis regarding long-term mortality (until December 31, 2020) for critically ill patients aged ≥ 70 years and discharged alive at index (*n* = 303)

**Variable**	**Prevalence** **n (%)**	**Unadjusted** **HR (95% CI)**	***p*-value**
CCI > 2	95 (31.4)	1.86 (1.43–2.42)	< 0.0001
Age; mean (SD)	81.7 (6.3)	1.07 (1.05–1.09)	< 0.0001
Female sex	138 (45.5)	1.06 (0.82–1.35)	0.66
ICU/cICU	59 (19.5)	0.88 (0.64–1.20)	0.42
LOS > 7 days	177 (58.4)	1.84 (1.42–2.37)	< 0.0001
Diagnosis at discharge			
Pneumonia	64 (21.1)	1.57 (1.17–2.09)	0.002
Heart failure	26 (8.6)	1.44 (0.94–2.22)	0.10
Atrial fibrillation	21 (6.9)	0.42 (0.24–0.76)	0.004
COPD	19 (6.3)	1.45 (0.91–2.32)	0.12
Urosepsis	23 (7.6)	0.80 (0.49–1.29)	0.33
Symptoms on admissiona			
Dyspnea	148 (48.8)	1.48 (1.16–1.89)	0.002
Unconsciousness	33 (10.9)	1.24 (0.84–1.81)	0.28
Chest pain	39 (12.9)	0.72 (0.49–1.06)	0.10
Seizure attack	15 (5.0)	1.03 (0.58–1.84)	0.91
Vomiting	19 (6.3)	0.94 (0.58–1.54)	0.81
Vital signs on admission			
Obstructive airway	17 (5.6)	1.05 (0.62–1.76)	0.87
Hypoxiab (5)c	153 (51.3)	1.99 (1.55–2.56)	< 0.0001
Hypotensiond (2)	40 (13.3)	1.05 (0.73–1.51)	0.78
Respiratory rate (br/min), ≤ 8/ ≥ 30 (35)	152 (56.7)	1.58 (1.20–2.07)	0.001
Heart rate (bpm), ≥ 130/ ≥ 150e (1)	69 (22.8)	0.56 (0.41–0.77)	0.0003
RLS > 3	38 (12.5)	1.08 (0.74–1.56)	0.69
Ongoing seizure attack	13 (4.3)	1.09 (0.60–2.00)	0.77
Signs of infection	111 (36.6)	0.95 (0.74–1.23)	0.71
Admission time point			
Workday 8 am—8 pm	143 (47.2)	0.73 (0.57–0.94)	0.01

Three hundred three patients were included in the analysis. There were two hundred fifty-four deaths

*CCI* Charlson Comorbidity Index, *ICU* intensive care unit, *cICU* coronary intensive care unit, *LOS* length of stay, *COPD* chronic obstructive pulmonary disease, *br/min* breaths per minute, *bpm* beats per minute, *RLS* Reaction Level Scale

^a^Five most commonly reported main symptoms in the ambulance

^b^Oxygen saturation < 90%

^c^Number missing

^d^Systolic blood pressure < 90 mmHg

^e^Regular/irregular

The following variables, excluding symptoms and vital signs on admission, were identified as independent predictors for long-term mortality, presented in order in terms of magnitudes of the HRs: CCI > 2 (HR 1.90, 95% CI 1.46–2.48), LOS > 7 days (HR 1.72, 95% CI 1.32–2.23), discharge diagnosis of pneumonia (HR 1.65, 95% CI 1.24–2.21) and age (HR 1.08 per increasing year, 95% CI 1.05–1.10) (all *p* < 0.001), see Table 5. In addition, when including symptoms and vital signs on admission to the ED, hypoxia was also identified as a predictor (HR 1.70, 95% CI 1.30–2.22), together with all the other factors (all *p* < 0.05). Furthermore, in sensitivity analyses where the CCI was used alternatively as an ordinal (i.e. not dichotomized) variable, the results were similar (data not shown, all *p* < 0.05).

**Table 5:** Multivariable analysis of predictors of long-term mortality (until December 31, 2020) for critically ill patients aged ≥ 70 years and discharged alive at index (*n* = 303)

Variable	Multivariable HR (95% CI)	*p*-value
CCI > 2	1.90 (1.46–2.48)	< 0.0001
Age; per year	1.08 (1.05–1.10)	< 0.0001
LOS > 7 days	1.72 (1.32–2.23)	< 0.0001
Diagnosis at discharge Pneumonia	1.65 (1.24–2.21)	0.0007

Three hundred three patients were included in the analysis. There were two hundred fifty-four deaths

### Re-hospitalizations

Among older adults alive 12 months post-discharge, the mean numbers of re-hospitalizations within 12 months were 1.5 (SD 1.8) for patients with a CCI of > 2 (*n* = 53), compared with 1.3 (SD 1.6) for patients with a CCI of ≤ 2 (*n* = 140), *p* = 0.31. The corresponding mean total post-discharge LOS were 15.3 days (SD 21.3) and 11.2 days (SD 18.6) respectively, *p* = 0.16.

Of those discharged alive (*n* = 303), 18.9% (18/95) of patients with CCI > 2 and 28.4% (59/208) of patients with CCI 0–2 were free from both rehospitalization and death 12 months after discharge, *p* = 0.04. The corresponding KM estimates regarding rehospitalization only (censoring non-rehospitalized patients at time of death) were 25.7% and 34.6% respectively (*p* = 0.09).

## Discussion

The results of this study show that, among critically ill older adults admitted to an ED and discharged alive from hospital, the following factors were predictive, after multivariable adjustment, of long-term mortality, when all patients were followed for 6.5–7.5 years post-discharge: CCI > 2, LOS > 7 days, hypoxia on admission, discharge diagnosis pneumonia, and age. The following factors were predictive of mortality at 30 days after ED admission: unconsciousness on admission, hypoxia on admission and age. For patients scoring CCI > 2 there was an almost two-fold long-term increase in the risk of death compared with those with lower CCI scores.

It is a strength of this study that the primary outcome analysis, i.e. that of long-term mortality, was based on a follow-up of 6.5–7.5 years post-discharge, where survival information was complete for all but one patient (who emigrated). This was done via a comprehensive state agency register, which constitutes a reliable source of mortality data. Another important strength is that different time perspectives were applied, i.e. the analyses focused on predictors of both short-term and long-term mortality. This is clinically relevant when it comes to individualized care planning, which should take account of risk prediction in different time perspectives. The potential adverse effects of many interventions are immediate, whereas the benefits of preventive interventions accumulate over time. It is therefore reasonable that clinical priorities and decision-making vary to some extent with life expectancy.

Of all patients identified as critically ill in the ED, two thirds were aged ≥ 70 years. Most older adults had a large comorbidity burden, the most common conditions being cardiovascular disease, diabetes, COPD and dementia, and one third scored > 2 on the CCI. The in-hospital mortality was approximately 25 per cent. These results harmonize with the results of previous studies of severely ill older adults [2, 6, 12]. The total long-term mortality in this population of older adults admitted to the ED was 88 per cent. A high mortality might have been expected considering the baseline characteristics of this severely ill population.

The most common discharge diagnoses were pneumonia, heart failure, urosepsis, COPD and atrial fibrillation. The mean LOS was high, more than double the mean LOS registered in Swedish hospitals [30], reflecting these patients’ severity of illness, their total morbidity burden and, subsequently, their severe health status and high care needs. LOS was also identified as an important marker of long-term mortality. A low percentage of critically ill older adults was treated in the ICUs. There might be different reasons for this finding, such as underuse or an estimated poor prognosis independent of possible interventions connected with ICU care.

The majority of the patients discharged and still alive after one year had at least one re-hospitalization within this year, which is in line with previous findings [7]. These study patients’ one-year total length of hospital stays was long. In unadjusted analysis, there were no significant differences regarding the impact of the CCI on re-hospitalizations and total LOS, probably because of the very high one-year mortality among those with CCI > 2. Of those discharged alive, a significantly lower percentage of patients scoring CCI > 2 was free from both rehospitalization and death 12 months after discharge.

There are some limitations and points to discuss in connection with our study. To the best of our knowledge, there is no generally accepted agreement on how to define critically ill patients or patients suffering from time-sensitive conditions [31]. In this study, critically ill patients were defined according to the RETTS, which is the system that was used for risk assessment in most EDs in Sweden at the time of patient inclusion. In the prehospital setting, the system has been associated with both over- and undertriage [32]. However, among patients aged > 65 years, specificity is increasing at the expense of decreasing sensitivity [32]. Quality of life (QoL) was not measured. We acknowledge the importance of QoL as a relevant outcome measurement for a population of older adults, although life expectancy is of the utmost importance for decision-making in an elderly population. This investigation did not include frailty as a predictor of risk. Frailty, a marker of biological age, could be a relevant confounder regarding risk prediction, when assessed with an established instrument. However, we focused on the burden of diagnoses, i.e. the comorbidity burden measured using the CCI, extracted from the medical records, which might be easily implemented in the ED. It should be noted that patients treated for cardiac arrest, in need for acute percutaneous coronary intervention (PCI) or being included in the acute stroke fast track were not included in the present study, since they were treated via separate, specific acute pathways. However, the large majority of all patients in the ED with acute cerebrovascular disease or acute coronary syndrome were included in the analysis. The NU Hospital Group was the only hospital serving the community. Primary care records were not included and we can not exclude the possibility that some patients were treated in a different hospital post-discharge. However, the judgement of the primary outcome, all-cause post-discharge death, was not dependent on health care records, but on a comprehensive and centralized state agency register. Moreover, critically ill patients constitute a particular cohort, and our results can not be generalized to all patients over the age of 70.

In future studies of risk predictors in critically ill older adults admitted to EDs, the assessment of frailty using an established instrument can be recommended. The predictive power of the CCI could then be compared with a frailty assessment of older adults. Furthermore, these studies should aim at including predictors of QoL. The possible effect of CGA on critically ill older adults in both the acute and post-acute phase should also be investigated.

## Conclusion

The results of this study show that, among critically ill older adults admitted to an ED and discharged alive, the following factors were independently predictive of long-term mortality: CCI > 2, LOS > 7 days, hypoxia on admission, discharge diagnosis of pneumonia, and age. For patients scoring CCI > 2 there was an almost two-fold long-term increase in the risk of death compared with those with lower CCI scores. The following factors were predictive of mortality at 30 days after ED admission: unconsciousness on admission, hypoxia and age. These data might be clinically relevant when it comes to individualized care planning, which should take account of risk prediction in different time perspectives.

## Data Availability

The datasets used and/or analysed during the current study are available from the corresponding author on reasonable request.

## Abbreviations

CCI: Charlson Comorbidity Index
CGA: Comprehensive geriatric assessment
COPD: Chronic obstructive pulmonary disease
ED: Emergency department
ESS: Emergency Signs and Symptoms
GCS: Glasgow Coma Scale
HR: Hazard ratio
ICU: Intensive care unit
KM: Kaplan–Meier
LOS: Length of stay
MEC: Medical Emergency Care
PCI: Percutaneous coronary intervention
RETTS: Rapid Emergency Treatment Triage System
RLS: Reaction Level Scale
SPAR: State’s Personal Address Register
VS: Vital signs
